# Comparison of Adverse Events Among Angiotensin Receptor Blockers in Hypertension Using the United States Food and Drug Administration Adverse Event Reporting System

**DOI:** 10.7759/cureus.81912

**Published:** 2025-04-08

**Authors:** Toru Ogura, Chihiro Shiraishi

**Affiliations:** 1 Department of Biostatistics, Clinical Research Support Center, Mie University Hospital, Tsu, JPN; 2 Department of Emergency and Disaster Medical Pharmacy, Fukuoka University, Fukuoka, JPN

**Keywords:** antihypertensive therapy, drug safety, drug-specific variations, personalized medicine, pharmacovigilance, retrospective analysis

## Abstract

Background

Angiotensin receptor blockers (ARBs) are pivotal in hypertension management. Despite sharing a common mechanism of blocking angiotensin II receptors, ARBs exhibit varying pharmacokinetic and pharmacodynamic properties that influence safety profiles. ARBs have been linked to adverse events (AEs) across multiple organ systems, including skin (e.g., angioedema), neurological (e.g., dizziness), and cardiovascular disorders (e.g., hypotension). Understanding these differences is essential for optimizing clinical decision-making.

Objectives

This study compared AE profiles of seven ARBs in patients with hypertension using data from the United States Food and Drug Administration Adverse Event Reporting System (FAERS). To enhance result reliability and control for confounding factors, only cases where ARBs were explicitly indicated for hypertension treatment were included. FAERS is a valuable post-marketing surveillance tool that captures spontaneous AE reports, although it has limitations such as reporting bias. The findings aim to generate hypotheses regarding ARB-associated AEs for future research using robust study designs.

Methods

A retrospective analysis of FAERS data between 2004 and 2024 was conducted. Patients prescribed ARBs for hypertension were included, while those with missing prescription indications or alternative uses were excluded. Reporting odds ratio (ROR) and adjusted ROR (aROR) were calculated to compare reporting proportions (RPs) among ARBs, with each ARB sequentially used as the reference in pairwise comparisons. The Bonferroni correction addressed multiple comparisons, with an adjusted significance level of 0.05/21=0.0024. For aROR calculations, cases with unknown values in adjustment variables were excluded. To enhance robustness, results were considered significant only when both ROR and aROR showed significance.

Results

Using losartan as the reference, valsartan, irbesartan, candesartan, telmisartan, and olmesartan demonstrated significantly lower RPs for skin disorders. For instance, the RP for skin disorders was 10.6 for losartan compared to 6.1 for valsartan (ROR: 0.545, p<0.0001; aROR: 0.648, p<0.0001) and 4.2 for olmesartan (ROR: 0.368, p<0.0001; aROR: 0.412, p<0.0001). Conversely, valsartan and olmesartan exhibited significantly higher RPs for cardiovascular disorders, with 23.7 for valsartan (ROR: 1.574, p<0.0001; aROR: 1.570, p<0.0001) and 19.0 for olmesartan (ROR: 1.186, p<0.0001; aROR: 1.278, p<0.0001) compared to 16.5 for losartan. Similar trends were observed when other ARBs were used as references, revealing a heterogeneous distribution of AE profiles among the seven ARBs.

Conclusions

This study reveals distinct AE patterns among ARBs in hypertension management. No single ARB exhibited universally favorable safety profiles across all AE categories, emphasizing the need for personalized prescribing. When selecting an ARB, prescribers should consider patient-specific risk factors and comorbidities. For instance, patients with a history of skin disorders may benefit from ARBs other than losartan. Conversely, patients with elevated cardiovascular risk may require closer monitoring when prescribed valsartan, including more frequent follow-up visits or additional cardiovascular diagnostics to detect early AEs. These findings enable healthcare providers to tailor ARB selection and monitoring strategies to optimize efficacy and safety in hypertension treatment.

## Introduction

Angiotensin receptor blockers (ARBs) have revolutionized hypertension management since the introduction of losartan in 1995 [1]. These medications selectively inhibit the angiotensin type 1 (AT1) receptor, preventing angiotensin II from binding and thereby mitigating its vasoconstrictive and aldosterone-secreting effects [2]. This mechanism promotes vasodilation, reduces sodium retention, and ultimately, lowers blood pressure. Following losartan's approval, several ARBs have been developed, each with distinct pharmacological properties and dosing regimens [3]. For instance, valsartan is typically prescribed at 80-160 mg once daily, while irbesartan and candesartan are administered at 150-300 mg and 16-32 mg once daily, respectively. Telmisartan, notable for its extended half-life of approximately 24 hours, is dosed at 40-80 mg once daily. More recent additions to the ARB class include olmesartan and azilsartan, generally dosed at 20-40 mg and 40-80 mg once daily, respectively. Although ARBs share a common mechanism of action, they exhibit diverse pharmacokinetic profiles, binding affinities for the AT1 receptor, and unique pharmacodynamic characteristics [4]. These differences influence their efficacy, duration of action, and potential ancillary benefits beyond blood pressure regulation. For example, telmisartan’s extended half-life may offer advantages in patients requiring sustained blood pressure control, while losartan’s active metabolite contributes to its unique pharmacodynamic profile [1,3]. Such pharmacokinetic and pharmacodynamic differences may also impact the safety profiles of individual ARBs, as previous studies have suggested links between these characteristics and adverse event (AE) risks.

ARBs are associated with a range of AEs affecting various organ systems, including skin, electrolyte, respiratory, neurological, cardiovascular, general, renal, and gastrointestinal disorders [5,6]. Among these, the most clinically relevant AEs include severe events such as hyperkalemia, renal dysfunction, and angioedema, due to their potential for life-threatening complications. Additionally, AEs (e.g., hypotension and rash) that frequently lead to treatment discontinuation are critical for optimizing patient adherence and outcomes. While mild-to-moderate AEs (e.g., dizziness and headache) are less critical from a clinical perspective, they may still impact patient quality of life and long-term adherence to therapy. While many AEs are similar across the seven ARBs, some variations exist among individual agents within this class [7]. For clinicians, navigating this complex landscape of potential AEs is critical to selecting the most appropriate ARB for each patient. This complexity arises primarily from the unique pharmacological properties of individual ARBs, including differences in pharmacokinetics, binding affinities for the AT1 receptor, and pharmacodynamic characteristics. The pharmacological distinctions, combined with patient-specific factors such as comorbidities and concomitant medications, create a nuanced landscape that clinicians must navigate. Additionally, the complexity is further compounded by challenges in post-marketing surveillance, including potential inconsistencies in AE reporting and the lack of comprehensive head-to-head comparative studies among all ARBs. Current clinical guidelines often discuss ARBs as a homogeneous class, without accounting for these nuanced differences among individual agents. For example, guidelines may recommend ARBs generically for hypertension management without distinguishing their specific safety profiles or pleiotropic effects This generalized approach may result in suboptimal treatment outcomes or unnecessary AEs in certain patients [3]. Consequently, there is a pressing need for comprehensive studies that elucidate the specific AE profiles of each ARB while addressing gaps such as the lack of real-world evidence or head-to-head trials.

Given the differences in efficacy, safety profiles, and potential pleiotropic effects among ARBs, detailed comparative studies are essential to guide optimal drug selection in clinical practice. Previous research utilizing data from the United States Food and Drug Administration Adverse Event Reporting System (FAERS) has often focused on specific AEs, included only a limited number of ARBs in its analyses, or lacked rigorous statistical adjustments to account for confounding factors [8-10]. Such limitations may restrict the applicability of findings to broader clinical decision-making contexts. In contrast, this study differentiates itself by analyzing seven ARBs comprehensively over a 20-year period using FAERS data, employing robust statistical methods such as Bonferroni corrections to ensure reliability and minimize bias. In reality, clinicians must consider a wide spectrum of potential AEs when choosing an ARB rather than focusing on isolated events. Current guidelines that treat ARBs as a uniform class risk neglecting nuanced differences between individual agents.

This study seeks to address these gaps in understanding the safety profiles of ARBs by comprehensively analyzing reported AEs associated with seven ARBs used in hypertension management. Through a retrospective analysis of FAERS data between 2004 and 2024, we employed rigorous statistical methods for multiple comparisons among all seven ARBs. The primary focus of this study is on safety profiles, with efficacy indicators assessed indirectly as secondary considerations. By focusing on results that demonstrated statistical significance in both reporting odds ratio (ROR) and adjusted ROR (aROR) analyses, we aimed to ensure robust findings that are consistent across different analytical methods [11]. Given the inherent limitations of spontaneous reporting data, including underreporting, selective reporting, and competition bias, the primary goal of this study is to generate reliable hypotheses regarding ARB-associated AEs. These hypotheses can be validated through future research using more robust study designs, such as clinical trials or observational cohort studies. By focusing exclusively on hypertension management, this study aims to provide clinicians with actionable insights into the relative safety profiles of these drugs while supporting hypothesis-driven research that may ultimately enhance personalized treatment strategies.

## Materials and methods

Data source

This study utilized the FAERS database, a comprehensive repository of AE reports submitted quarterly since 2004. Originally known as the Adverse Event Reporting System (AERS), the database transitioned to FAERS in the fourth quarter of 2012 (2012Q4). Data files from AERS and FAERS (aers_ascii_yyyyQq.zip and faers_ascii_yyyyQq.zip, where yyyy denotes the year and q the quarter) were accessed from the official FAERS website on January 31, 2025. To ensure consistency between databases, differences in variable names and structures were harmonized based on their respective documentation. For instance, {ISR} in AERS was mapped to {primaryid} in FAERS for report identification, while {CASE} was mapped to {caseid} for case identification. Throughout this manuscript, we use curly braces to denote variable names as they appear in the FAERS database. These mappings were crucial for maintaining data continuity and enabling accurate tracking of reports across the entire study period. Additionally, we accounted for changes introduced in FAERS, such as the inclusion of the {caseversion} variable and updates made in 2014Q3, which included renaming {gndr_cod} to {sex} and adding {prod_ai} for product active ingredients. These variable name changes did not affect the underlying data structure or require reclassification of earlier datasets. Our standardization process ensured that data from all periods were consistently coded and analyzed. From the FAERS database, five key data files were selected for analysis: patient demographic and administrative information (DEMOyyQq.txt, where yy represents the last two digits of the year), drug information (DRUGyyQq.txt), AE information (REACyyQq.txt), drug therapy start and end dates (THERyyQq.txt), and indications for use (INDIyyQq.txt).

The FAERS system employs a version control mechanism whereby updates to reports increment the version number {caseversion} without overwriting previous data. For this study, only the highest {caseversion} number was used for each report to ensure data accuracy. In AERS, where {caseversion} was not available, unique numbers for identifying such as {ISR} and case identification number {CASE} were used to determine equivalent cases. Several preprocessing steps were undertaken to standardize and clean the data. The units of the {age} variable were converted to years, and those of the {weight} variable were converted to kilograms. Inconsistent or unexpected inputs for variables such as {sex}, {age}, {weight}, and reporter's country {reporter_country} were addressed systematically. Additionally, line breaks missing in certain AERS files were manually inserted at lines 322,967, 247,896, and 446,738 of DRUG11Q2.txt, DRUG11Q3.txt, and DRUG11Q4.txt, respectively, to ensure proper data parsing. Without these line break corrections, critical drug-event associations could have been misrepresented or excluded from the analysis due to structural errors in the dataset. Importantly, this preprocessing step did not alter the content of the data but restored its intended structure to ensure accurate parsing and reliable analysis. Detailed descriptions of these preprocessing procedures are provided in Appendix A. By harmonizing data across AERS and FAERS databases and addressing inconsistencies through rigorous preprocessing methods, this study ensured a high level of data quality and reliability for subsequent analyses.

Study design

This study was designed to investigate AEs associated with ARB use in hypertensive patients. The inclusion criteria encompassed patients who received ARBs for hypertension between 2004Q1 and 2024Q4. To identify eligible cases, different methods were employed for the AERS and FAERS data phases. For the AERS data, comprehensive searches were conducted using both generic and brand names of ARBs within the {drugname} variable, as delineated in Appendix B. For the FAERS data, the introduction of the {prod_ai} variable allowed for a identification of ARBs by their generic names. From these records, only cases explicitly indicating hypertension as the reason for ARB use were retained, as identified in the {indi_pt}. Specific terms used to define patients with hypertension are provided in Appendix C.

To ensure the accuracy of AE attribution and refine the database, specific exclusion criteria were applied. Patients were excluded only when it could be reliably determined that ARBs were not being administered at the time of the AE. This determination was based on three key date fields: {start_dt} (therapy start or restart date), {event_dt} (date the AE occurred or began), and {end_dt} (therapy stop date). Cases with missing or incomplete date information were retained at this stage to preserve the database's comprehensiveness. Further refinement was conducted using the {role_cod} variable, which identifies whether an ARB was likely responsible for the reported AE. Patients who had been administered multiple types of ARBs were excluded to avoid confounding effects from combination therapy. Additionally, only cases where ARBs were identified as the primary suspect drug for the reported AE were included. This determination relied on the {role_cod} field, where cases marked as "PS" (primary suspect) were selected. By focusing exclusively on monotherapy cases where ARBs were prescribed for hypertension and identified as the primary suspect drug, this study aimed to reduce confounding influences from comorbidities or concomitant medications. Duplicate entries were addressed by excluding cases with different identification numbers but identical information across multiple fields, including drug name, sex, age, weight, country, AE occurrence date, administration start date, administration end date, {role_cod}, and AE name. FAERS data often contains multiple reports for the same patient submitted by different healthcare professionals. Entries with identical patient background variables across these fields were considered duplicate reports rather than coincidental matches. We acknowledge that our strict criteria for hypertension case identification and duplicate removal may have excluded some relevant cases. However, this approach was chosen to ensure high specificity in our cohort and minimize subjective interpretations. The exclusion of patients on multiple ARBs, while potentially introducing selection bias, allowed us to focus on the effects of individual ARBs in a more homogeneous population. Future studies may consider broader inclusion criteria to capture a wider range of hypertension cases and ARB use patterns. This approach ensured that our analysis captured ARB-specific safety profiles while minimizing unnecessary exclusions due to incomplete data and reducing duplicate report bias.

The primary endpoint was the occurrence of AEs. These events were described using the Medical Dictionary for Regulatory Activities (MedDRA) preferred terms {pt}. The FAERS database is updated quarterly and utilizes the most current version of MedDRA available at each release. To maintain data integrity, original spellings of MedDRA preferred terms were preserved as provided in the FAERS database, including instances where British English spellings were used. AEs were categorized into nine classifications, as detailed in Appendix D. Among these, efficacy indicators were defined as AEs such as blood pressure inadequately controlled, drug ineffective, and hypertension. These events were considered potential markers of ARB effectiveness, based on the premise that they are less likely to occur with effective ARB therapy and more likely to occur with suboptimal treatment. It is important to note that the FAERS database does not provide direct measures of drug efficacy. However, analyzing the characteristics of reported AEs enables an indirect assessment of efficacy indicators. While this approach offers insights into the relative effectiveness of different ARBs, it must be interpreted cautiously due to the inherent limitations of spontaneous reporting data. The indirect nature of this assessment highlights the need for validation through clinical trials and other robust study designs to draw more definitive conclusions about ARB effectiveness.

Statistical analyses

Continuous variables were summarized as median with first and third quartiles. Categorical variables were summarized as frequency with reporting proportion (RP), calculated as RP=(number of patients reported in the category of interest)/(the total number of patients reported receiving a particular ARB)×100 [12]. To compare AE profiles among ARBs, we employed two primary statistical measures: ROR and aROR. The ROR and its confidence interval (CI) were calculated using univariate binomial logistic regression analysis. The aROR and its CI were calculated using multivariate binomial logistic regression analysis, adjusting for potential confounding factors. The adjusted variables in the aROR calculation included {age}, {sex}, and {reporter_country}. Given that the United States had the highest number of reports, we treated {reporter_country} as binary data (the United States vs. other countries) in both univariate and multivariate analyses. Patients with unknown values for any of the three adjustment variables were excluded from the aROR calculation. While methods for imputing unknown values exist, the limited number of patient background variables in the FAERS database makes high-accuracy imputation challenging. To maintain data integrity, we opted for exclusion rather than imputation in these cases. We conducted 21 pairwise comparisons, sequentially setting each ARB as the reference. To address the issue of multiple comparisons, we applied the Bonferroni correction, adjusting the significance level to 0.05/21=0.0024 [13]. Consequently, p<0.0024 was considered statistically significant. The corresponding confidence level for the CIs was set at 99.76%, ensuring consistency between the statistical hypothesis test and CI interpretation. This approach guarantees that when a result is statistically significant, the 99.76% CI will not include one. The choice of ROR and aROR over alternative statistical methods was based on several considerations. First, ROR and aROR allow for easier adjustment of significance levels in multiple comparisons, which was essential for our study design involving 21 pairwise comparisons. Second, the combination of ROR and aROR provided a balance between data inclusivity and confounder adjustment, ensuring robust results while maintaining statistical rigor. Third, ROR is widely used in pharmacovigilance studies, which facilitates comparability with other research findings and enhances the interpretability of our results within the broader context of drug safety analysis.

It is crucial to note that FAERS data only include reports of AE occurrences, with no reports of zero AEs. Consequently, we could not calculate the true incidence rate of each AE. This limitation contrasts with observational cohort studies and clinical trials, which typically provide denominator data (e.g., total number of exposed patients) for calculating incidence rates. As a result, while FAERS allows for studies of various populations, its lack of denominator data restricts direct comparability with these study designs. Observational cohort studies and clinical trials offer precise incidence rates but are often limited by smaller sample sizes, stricter inclusion criteria, and shorter follow-up durations. In contrast, FAERS captures real-world AE reporting across heterogeneous populations, albeit with inherent biases such as underreporting and selective reporting. To distinguish our methods from conventional statistical techniques, we prefixed "reporting" to the names of our statistical analysis methods, following the precedent set by previous studies utilizing FAERS data. All statistical analyses were performed using software R version 4.4.1 (R Foundation for Statistical Computing, Vienna, Austria).

## Results

Patient background

**Figure 1:**
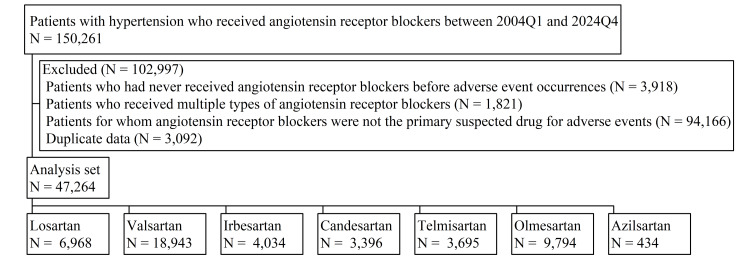
Flowchart of patients with hypertension who received ARBs ARB: angiotensin receptor blocker

FAERS data between 2004Q1 and 2024Q4 were analyzed, initially identifying 150,261 patients receiving ARBs for hypertension management. After applying rigorous exclusion criteria, 102,997 patients were excluded, resulting in a final cohort of 47,264 patients. This substantial reduction in sample size underscores the commitment to data integrity and minimizing confounding factors while retaining a cohort sufficiently large for robust statistical analyses. The distribution of patients across the seven ARBs is illustrated in Figure 1. The cohort comprised 6,968 patients receiving losartan, 18,943 receiving valsartan, 4,034 receiving irbesartan, 3,396 receiving candesartan, 3,695 receiving telmisartan, 9,794 receiving olmesartan, and 434 receiving azilsartan. A detailed summary of patient demographics and baseline characteristics for each ARB is presented in Table 1. The higher RP of female patients across all ARBs could be attributed to several factors [14]. Biologically, females have a higher prevalence of hypertension after menopause. Additionally, females are more likely to seek medical care and report AEs. Prescribing patterns may also play a role, as some studies suggest that females are more frequently prescribed certain antihypertensive medications. Similar trends in female predominance have been observed in other pharmacovigilance databases and real-world studies for ARBs, further supporting this observation [14,15]. However, we acknowledge that reporting bias in the FAERS database could also contribute to this finding. The median age ranged from 65 to 71 years across the seven ARBs, indicating that these medications are predominantly used in older adult populations. Weight data showed a high RP of unknown values across all ARBs, limiting interpretability for weight-related findings. The RP of unknown weight values varied considerably across ARBs, ranging from 50.6 for candesartan to 82.0 for azilsartan. This variation could reflect differences in reporting practices or patient populations across different ARBs. Among cases with available weight data, median weights ranged from 70 to 80 kg for most ARBs; however, azilsartan users demonstrated a notably higher median weight of 88.1 kg. Including weight as an adjustment factor in the aROR calculation would have significantly reduced our sample size and potentially compromised the representativeness of our analysis. Therefore, weight was not included as an adjustment variable in aROR. Nevertheless, we recognize that weight can be an important factor in ARB-related AEs and emphasize that future research should focus on addressing this limitation by using data sources with more complete weight information. Geographically, the majority of AE reports originated from the United States for all ARBs except irbesartan and candesartan. Differences in RPs across countries may be influenced by various factors, including regulatory actions, drug recalls, or cultural differences in AE reporting practices. For instance, mandatory reporting requirements in some regions or heightened awareness due to regulatory communications may lead to increased reporting rates. Irbesartan had a high RP from France (31.8), which may reflect regional prescribing preferences or market penetration; irbesartan has been widely used in France since its introduction [16]. Candesartan showed a more globally distributed pattern of reports. This global distribution may be attributed to several factors, including its development in Japan, widespread use in international clinical trials, and variations in market introduction timing across countries. For valsartan, while the United States had the highest RP at 19.0, it was not markedly higher than other countries. Notably, Mexico (9.9) and Brazil (10.2) also contributed substantial RPs. In Brazil particularly, valsartan has been widely used in general practice as both monotherapy and combination therapy [17]. Local regulatory actions or post-marketing surveillance studies may have also contributed to increased reporting from these countries.

**Table 1:** Summary of patient background Age and weight are summarized as median and first and third quartiles. Other data are summarized as frequencies (RP). Q1: first quartile; Q3: third quartile; RP: reporting proportion

	Losartan	Valsartan	Irbesartan	Candesartan	Telmisartan	Olmesartan	Azilsartan
	N=6,968	N=18,943	N=4,034	N=3,396	N=3,695	N=9,794	N=434
Sex							
Female, n (RP)	3,749 (53.8)	10,565 (55.8)	2,165 (53.7)	1,926 (56.7)	1,963 (53.1)	2,790 (28.5)	126 (29.0)
Male, n (RP)	2,635 (37.8)	7,575 (40.0)	1,418 (35.2)	1,338 (39.4)	1,492 (40.4)	2,125 (21.7)	100 (23.0)
Unknown, n (RP)	584 (8.4)	803 (4.2)	451 (11.2)	132 (3.9)	240 (6.5)	4,879 (49.8)	208 (47.9)
Age (year)							
Median	67.5	69.0	71.0	69.0	68.0	68.0	65.0
Q1-Q3	58.0-76.0	60.0-78.0	61.0-80.0	58.0-78.0	58.0-76.0	58.0-76.0	57.0-71.0
Unknown, n (RP)	1,850 (26.5)	6,065 (32.0)	811 (20.1)	395 (11.6)	1,149 (31.1)	5,766 (58.9)	282 (65.0)
Weight (kg)							
Median	79.1	73.5	74.9	73.0	70.0	76.4	88.1
Q1-Q3	65.8-92.2	62.0-88.0	64.0-88.0	61.2-85.0	58.3-84.5	63.5-90.7	70.4-104.1
Unknown, n (RP)	3,692 (53.0)	11,993 (63.3)	2,411 (59.8)	1,717 (50.6)	2,443 (66.1)	7,678 (78.4)	356 (82.0)
Country							
Canada, n (RP)	134 (1.9)	434 (2.3)	131 (3.2)	134 (3.9)	128 (3.5)	147 (1.5)	6 (1.4)
Mexico, n (RP)	2 (0.0)	1,871 (9.9)	164 (4.1)	24 (0.7)	12 (0.3)	16 (0.2)	1 (0.2)
United States, n (RP)	3,556 (51.0)	3,601 (19.0)	826 (20.5)	433 (12.8)	896 (24.2)	4,913 (50.2)	382 (88.0)
Argentina, n (RP)	2 (0.0)	715 (3.8)	3 (0.1)	0 (0.0)	3 (0.1)	1 (0.0)	0 (0.0)
Brazil, n (RP)	163 (2.3)	1,932 (10.2)	46 (1.1)	506 (14.9)	68 (1.8)	726 (7.4)	0 (0.0)
China, n (RP)	61 (0.9)	640 (3.4)	157 (3.9)	11 (0.3)	31 (0.8)	28 (0.3)	2 (0.5)
Japan, n (RP)	176 (2.5)	2,618 (13.8)	109 (2.7)	483 (14.2)	527 (14.3)	604 (6.2)	1 (0.2)
France, n (RP)	290 (4.2)	644 (3.4)	1,281 (31.8)	627 (18.5)	198 (5.4)	498 (5.1)	0 (0.0)
Germany, n (RP)	72 (1.0)	746 (3.9)	79 (2.0)	289 (8.5)	70 (1.9)	192 (2.0)	6 (1.4)
Italy, n (RP)	255 (3.7)	334 (1.8)	238 (5.9)	72 (2.1)	141 (3.8)	843 (8.6)	0 (0.0)
Spain, n (RP)	199 (2.9)	191 (1.0)	81 (2.0)	50 (1.5)	48 (1.3)	437 (4.5)	0 (0.0)
United Kingdom, n (RP)	560 (8.0)	140 (0.7)	176 (4.4)	148 (4.4)	29 (0.8)	61 (0.6)	0 (0.0)
South Africa, n (RP)	189 (2.7)	167 (0.9)	4 (0.1)	23 (0.7)	20 (0.5)	0 (0.0)	0 (0.0)
Others, n (RP)	913 (13.1)	1,839 (9.7)	497 (12.3)	382 (11.2)	376 (10.2)	747 (7.6)	36 (8.3)
Unknown, n (RP)	396 (5.7)	3,071 (16.2)	242 (6.0)	214 (6.3)	1,148 (31.1)	581 (5.9)	0 (0.0)

Adverse events

Table 2 provides a comprehensive summary of AE categories for each ARB, while Table 3 presents the ROR and aROR values for AE categories, using losartan as the reference. Although aROR is generally preferred due to its adjustment for confounding factors, its calculation was limited by substantial unknown values in the FAERS database. All 47,264 patients were utilized for ROR calculations. However, for aROR calculations, only 27,034 patients were used due to unknown values in certain variables. Specifically, there were 7,297 patients with unknown values for the {sex} variable, 16,313 patients with unknown values for the {age} variable, and 5,652 patients with unknown values for the {reporter_country} variable. Since aROR calculations exclude any patient with an unknown value in any of these three variables, the number of patients eligible for aROR analysis was significantly reduced. The substantial reduction in sample size for aROR analysis could potentially introduce bias. To address this concern and ensure robust findings, this study focused on ARBs exhibiting p<0.0024 for both ROR and aROR results. This approach helps mitigate the risk of false positives and ensures that our findings are consistent across both the full and reduced datasets. While methods for imputing unknown values exist, the limited number of patient background variables in the FAERS database makes high-accuracy imputation challenging. To maintain data integrity, we opted for exclusion rather than potentially introducing additional bias through imputation. The differences in significance between ROR and aROR results could be attributed to the reduced sample size in aROR calculations and the adjustment for these confounding factors. By focusing on ARBs that showed statistical significance in both ROR and aROR results, thereby increasing the reliability of our findings.

**Table 2:** Summary of AE category Data are summarized as frequencies (reporting proportions). If multiple AEs are reported for a patient, each AE is counted. Each patient is counted only once per AE category, regardless of the number of preferred terms experienced within that category. AE: adverse event

	Losartan	Valsartan	Irbesartan	Candesartan	Telmisartan	Olmesartan	Azilsartan
	N=6,968	N=18,943	N=4,034	N=3,396	N=3,695	N=9,794	N=434
Skin disorders	742 (10.6)	1,155 (6.1)	244 (6.0)	183 (5.4)	228 (6.2)	412 (4.2)	30 (6.9)
Electrolyte disorders	426 (6.1)	753 (4.0)	430 (10.7)	249 (7.3)	196 (5.3)	535 (5.5)	22 (5.1)
Respiratory disorders	517 (7.4)	1,199 (6.3)	197 (4.9)	262 (7.7)	241 (6.5)	321 (3.3)	29 (6.7)
Neurological disorders	1,161 (16.7)	2,948 (15.6)	436 (10.8)	472 (13.9)	478 (12.9)	1,449 (14.8)	80 (18.4)
Cardiovascular disorders	1,151 (16.5)	4,498 (23.7)	620 (15.4)	642 (18.9)	639 (17.3)	1,861 (19.0)	83 (19.1)
General disorders	770 (11.1)	2,432 (12.8)	367 (9.1)	376 (11.1)	292 (7.9)	1,481 (15.1)	60 (13.8)
Renal disorders	408 (5.9)	1,164 (6.1)	425 (10.5)	291 (8.6)	188 (5.1)	1,653 (16.9)	25 (5.8)
Gastrointestinal disorders	607 (8.7)	1,478 (7.8)	315 (7.8)	284 (8.4)	279 (7.6)	3,997 (40.8)	55 (12.7)
Efficacy indicators	788 (11.3)	2,122 (11.2)	368 (9.1)	388 (11.4)	399 (10.8)	778 (7.9)	51 (11.8)

**Table 3:** The ROR and aROR For the ROR and aROR, the reference categories for ARB type, sex, and country were set to losartan, female, and other countries, respectively. The sample sizes used for the ROR were 47,264 for ARB type, 39,967 for sex, 30,946 for age, and 41,612 for country. The sample size employed for the aROR remained consistent at 27,034. aROR: adjusted reporting odds ratio; CI: confidence interval; ROR: reporting odds ratio; ARB: angiotensin receptor blocker

	Univariate analysis		Multivariate analysis	
	ROR (99.76% of CI)	P-value	aROR (99.76% of CI)	P-value
Skin disorders				
Valsartan	0.545 (0.469-0.633)	<0.0001	0.648 (0.536-0.782)	<0.0001
Irbesartan	0.540 (0.428-0.682)	<0.0001	0.625 (0.474-0.825)	<0.0001
Candesartan	0.478 (0.369-0.619)	<0.0001	0.592 (0.441-0.797)	<0.0001
Telmisartan	0.552 (0.435-0.701)	<0.0001	0.623 (0.448-0.867)	<0.0001
Olmesartan	0.368 (0.304-0.447)	<0.0001	0.412 (0.312-0.545)	<0.0001
Azilsartan	0.623 (0.346-1.121)	0.0143	0.496 (0.189-1.300)	0.0271
Male	0.954 (0.843-1.079)	0.2446	1.028 (0.886-1.193)	0.5747
Age	0.990 (0.986-0.995)	<0.0001	0.993 (0.988-0.998)	<0.0001
United States	1.671 (1.480-1.888)	<0.0001	1.976 (1.689-2.311)	<0.0001
Electrolyte disorders				
Valsartan	0.636 (0.526-0.768)	<0.0001	0.481 (0.387-0.598)	<0.0001
Irbesartan	1.832 (1.475-2.276)	<0.0001	0.990 (0.770-1.272)	0.9008
Candesartan	1.215 (0.945-1.562)	0.0184	0.678 (0.513-0.897)	<0.0001
Telmisartan	0.860 (0.657-1.127)	0.0900	0.760 (0.542-1.066)	0.0139
Olmesartan	0.887 (0.724-1.087)	0.0741	0.847 (0.656-1.094)	0.0491
Azilsartan	0.820 (0.415-1.622)	0.3767	1.161 (0.394-3.422)	0.6750
Male	0.870 (0.759-0.997)	0.0019	0.869 (0.747-1.011)	0.0049
Age	1.045 (1.039-1.051)	<0.0001	1.041 (1.034-1.047)	<0.0001
United States	0.221 (0.181-0.268)	<0.0001	0.305 (0.242-0.385)	<0.0001
Respiratory disorders				
Valsartan	0.843 (0.714-0.995)	0.0018	0.929 (0.753-1.147)	0.2886
Irbesartan	0.641 (0.493-0.832)	<0.0001	0.718 (0.529-0.974)	0.0010
Candesartan	1.043 (0.821-1.326)	0.5925	1.086 (0.820-1.439)	0.3724
Telmisartan	0.871 (0.681-1.113)	0.0864	0.994 (0.712-1.386)	0.9537
Olmesartan	0.423 (0.339-0.528)	<0.0001	0.523 (0.384-0.712)	<0.0001
Azilsartan	0.893 (0.490-1.628)	0.5686	0.567 (0.173-1.860)	0.1467
Male	0.857 (0.753-0.975)	0.0003	0.825 (0.706-0.964)	0.0002
Age	0.998 (0.993-1.003)	0.2306	0.997 (0.992-1.002)	0.0822
United States	0.985 (0.862-1.125)	0.7312	1.148 (0.966-1.365)	0.0149
Neurological disorders				
Valsartan	0.922 (0.822-1.034)	0.0317	1.044 (0.899-1.212)	0.3833
Irbesartan	0.606 (0.505-0.727)	<0.0001	0.728 (0.584-0.907)	<0.0001
Candesartan	0.807 (0.675-0.966)	0.0003	0.988 (0.798-1.222)	0.8588
Telmisartan	0.743 (0.622-0.888)	<0.0001	0.994 (0.781-1.266)	0.9408
Olmesartan	0.868 (0.762-0.989)	0.0010	0.843 (0.695-1.021)	0.0068
Azilsartan	1.130 (0.766-1.667)	0.3381	0.807 (0.408-1.596)	0.3393
Male	0.639 (0.584-0.699)	<0.0001	0.584 (0.522-0.654)	<0.0001
Age	0.988 (0.985-0.992)	<0.0001	0.987 (0.983-0.991)	<0.0001
United States	1.813 (1.666-1.974)	<0.0001	1.804 (1.607-2.026)	<0.0001
Cardiovascular disorders				
Valsartan	1.574 (1.409-1.758)	<0.0001	1.570 (1.364-1.808)	<0.0001
Irbesartan	0.918 (0.778-1.082)	0.1141	0.900 (0.739-1.095)	0.1028
Candesartan	1.178 (0.999-1.390)	0.0026	1.121 (0.924-1.361)	0.0725
Telmisartan	1.057 (0.896-1.246)	0.3081	1.196 (0.956-1.496)	0.0151
Olmesartan	1.186 (1.046-1.344)	<0.0001	1.278 (1.074-1.521)	<0.0001
Azilsartan	1.195 (0.814-1.754)	0.1581	1.005 (0.506-1.999)	0.9810
Male	0.904 (0.837-0.976)	0.0001	0.903 (0.822-0.992)	0.0010
Age	1.005 (1.002-1.008)	<0.0001	1.004 (1.001-1.007)	0.0003
United States	0.784 (0.724-0.850)	<0.0001	0.863 (0.773-0.964)	0.0001
General disorders				
Valsartan	1.186 (1.037-1.355)	0.0001	1.225 (1.033-1.453)	0.0003
Irbesartan	0.806 (0.658-0.987)	0.0012	0.847 (0.664-1.081)	0.0387
Candesartan	1.002 (0.818-1.227)	0.9741	1.095 (0.865-1.386)	0.2443
Telmisartan	0.691 (0.555-0.859)	<0.0001	0.769 (0.567-1.041)	0.0084
Olmesartan	1.434 (1.241-1.657)	<0.0001	1.626 (1.334-1.981)	<0.0001
Azilsartan	1.291 (0.833-2.001)	0.0763	1.107 (0.518-2.367)	0.6831
Male	0.818 (0.743-0.901)	<0.0001	0.792 (0.704-0.891)	<0.0001
Age	0.999 (0.996-1.003)	0.6264	0.999 (0.995-1.003)	0.3368
United States	1.130 (1.028-1.242)	0.0001	1.167 (1.024-1.330)	0.0003
Renal disorders				
Valsartan	1.053 (0.879-1.261)	0.3869	0.899 (0.726-1.114)	0.1310
Irbesartan	1.893 (1.520-2.359)	<0.0001	1.416 (1.098-1.827)	<0.0001
Candesartan	1.507 (1.183-1.920)	<0.0001	1.103 (0.839-1.450)	0.2764
Telmisartan	0.862 (0.655-1.135)	0.1010	0.818 (0.573-1.169)	0.0878
Olmesartan	3.265 (2.740-3.890)	<0.0001	1.366 (1.067-1.748)	0.0001
Azilsartan	0.983 (0.516-1.873)	0.9348	1.929 (0.821-4.531)	0.0195
Male	1.473 (1.306-1.662)	<0.0001	1.446 (1.260-1.658)	<0.0001
Age	1.017 (1.012-1.022)	<0.0001	1.016 (1.011-1.021)	<0.0001
United States	1.113 (1.002-1.237)	0.0021	0.500 (0.414-0.605)	<0.0001
Gastrointestinal disorders				
Valsartan	0.887 (0.761-1.034)	0.0171	1.038 (0.857-1.257)	0.5557
Irbesartan	0.888 (0.712-1.106)	0.0998	0.943 (0.725-1.226)	0.4969
Candesartan	0.956 (0.761-1.202)	0.5525	1.112 (0.856-1.444)	0.2173
Telmisartan	0.856 (0.681-1.076)	0.0390	1.059 (0.780-1.436)	0.5702
Olmesartan	7.225 (6.260-8.339)	<0.0001	3.966 (3.264-4.818)	<0.0001
Azilsartan	1.521 (0.963-2.402)	0.0053	1.872 (0.943-3.715)	0.0055
Male	0.680 (0.611-0.757)	<0.0001	0.684 (0.603-0.777)	<0.0001
Age	0.999 (0.995-1.003)	0.5566	0.999 (0.995-1.004)	0.6291
United States	3.191 (2.936-3.469)	<0.0001	1.339 (1.171-1.532)	<0.0001
Efficacy indicators				
Valsartan	0.989 (0.865-1.132)	0.8092	1.114 (0.935-1.327)	0.0617
Irbesartan	0.787 (0.643-0.963)	0.0003	0.863 (0.673-1.106)	0.0716
Candesartan	1.012 (0.828-1.236)	0.8608	1.244 (0.984-1.574)	0.0046
Telmisartan	0.949 (0.779-1.157)	0.4252	1.209 (0.923-1.584)	0.0327
Olmesartan	0.677 (0.576-0.795)	<0.0001	1.121 (0.905-1.390)	0.1054
Azilsartan	1.044 (0.655-1.666)	0.7779	0.762 (0.321-1.813)	0.3411
Male	0.787 (0.711-0.870)	<0.0001	0.723 (0.639-0.819)	<0.0001
Age	0.990 (0.986-0.994)	<0.0001	0.989 (0.985-0.993)	<0.0001
United States	1.174 (1.062-1.297)	<0.0001	1.288 (1.127-1.473)	<0.0001

Compared to losartan, valsartan demonstrated significantly ROR<1 and aROR<1 for skin disorders (ROR: 0.545 (99.76% CI: 0.469-0.633), p<0.0001; aROR: 0.648 (99.76% CI: 0.536-0.782), p<0.0001). Similar trends were observed for other ARBs: irbesartan (ROR: 0.540 (99.76% CI: 0.428-0.682), p<0.0001; aROR: 0.625 (99.76% CI: 0.474-0.825), p<0.0001), candesartan (ROR: 0.478 (99.76% CI: 0.369-0.619), p<0.0001; aROR: 0.592 (99.76% CI: 0.441-0.797), p<0.0001), telmisartan (ROR: 0.522 (99.76% CI: 0.435-0.701), p<0.0001; aROR: 0.623 (99.76% CI: 0.448-0.867), p<0.0001), and olmesartan (ROR: 0.368 (99.76% CI: 0.304-0.447), p<0.0001; aROR: 0.412 (99.76% CI: 0.312-0.545), p<0.0001). Similar patterns of significantly ROR <1 and aROR <1 were observed in specific AE categories such as electrolyte disorders with valsartan (ROR: 0.636 (99.76% CI: 0.526-0.768), p<0.0001; aROR: 0.481 (99.76% CI: 0.387-0.598), p<0.0001); respiratory disorders with irbesartan (ROR: 0.641 (99.76% CI: 0.493-0.832), p<0.0001; aROR: 0.718 (99.76% CI: 0.529-0.974), p=0.0010) and olmesartan (ROR: 0.423 (99.76% CI: 0.339-0.528), p<0.0001; aROR: 0.523 (99.76% CI: 0.384-0.712), p<0.0001); and neurological disorders with irbesartan (ROR: 0.606 (99.76% CI: 0.505-0.727), p<0.0001; aROR: 0.728 (99.76% CI: 0.584-0.907), p<0.0001). Conversely, cardiovascular disorders demonstrated significantly ROR >1 and aROR <1 for valsartan (ROR: 1.574 (99.76% CI: 1.409-1.758), p<0.0001; aROR: 1.570 (99.76% CI: 1.364-1.808), p<0.0001) and olmesartan (ROR: 1.186 (99.76% CI: 1.046-1.344), p<0.0001; aROR: 1.278 (99.76% CI: 1.074-1.521), p<0.0001); general disorders in valsartan (ROR: 1.186 (99.76% CI: 1.037-1.355), p=0.0001; aROR: 1.225 (99.76% CI: 1.033-1.453), p=0.0003) and olmesartan (ROR: 1.434 (99.76% CI: 1.241-1.657), p<0.0001; aROR: 1.626 (99.76% CI: 1.334-1.981), p<0.0001); renal disorders in irbesartan (ROR: 1.893 (99.76% CI: 1.520-2.359), p<0.0001; aROR: 1.416 (99.76% CI: 1.098-1.827), p<0.0001) and olmesartan (ROR: 3.265 (99.76% CI: 2.740-3.890), p<0.0001; aROR: 1.366 (99.76% CI: 1.067-1.748), p=0.0001); and gastrointestinal disorders in olmesartan (ROR: 7.225 (99.76% CI: 6.260-8.339), p<0.0001; aROR: 3.966 (99.76% CI: 3.264-4.818), p<0.0001). For efficacy indicators, irbesartan and olmesartan showed significant ROR <1, but their aROR was not significant. Appendix E summarizes the results of both ROR and aROR using each of the other six ARBs sequentially as the reference, presented as one of three statistical test outcomes: both significantly ROR<1 and aROR<1, both significantly ROR >1 and aROR >1, and at least one of ROR or aROR is not significant. The fundamental properties of both ROR and aROR as odds ratio measures remain intact, enabling the derivation of detailed results, namely the ROR with its 99.76% CI and p-value, as well as the aROR with its 99.76% CI and p-value from Table 3 [18-20]. Upon reviewing the comprehensive results, it is evident that no single ARB consistently demonstrated low RPs across all AE categories. Instead, each ARB exhibited a heterogeneous profile of higher and lower RPs depending on the specific AE category. This variability highlights the complex safety profiles of these medications and underscores the importance of individualized treatment strategies tailored to each patient's unique needs and conditions. When selecting an ARB, it is essential to consider not only safety but also efficacy, necessitating a comprehensive evaluation that incorporates the results for efficacy indicators. For instance, olmesartan exhibited higher RPs for renal disorders and gastrointestinal disorders compared to the other six ARBs analyzed. However, it is equally important to note that olmesartan demonstrated lower RPs for efficacy indicators relative to the other ARBs. Lower RPs for efficacy indicators, such as blood pressure inadequately controlled or drug ineffective, suggest fewer reports of treatment failure, which may be indicative of higher drug effectiveness. However, it is important to note that this interpretation is based on indirect measures derived from spontaneous reporting data, which differ from direct efficacy assessments in clinical trials or observational cohort studies. For instance, clinical trials often use standardized blood pressure measurements or predefined endpoints to evaluate drug effectiveness more precisely. Despite these limitations, the observed lower RPs for efficacy indicators associated with olmesartan provide a hypothesis that it may offer superior therapeutic benefits compared to other ARBs. This finding underscores the importance of balancing safety and efficacy profiles when selecting an ARB for hypertension management.

These findings underscore the need for a balanced and holistic approach to ARB selection, taking into account both safety and efficacy profiles. Such an approach ensures that treatment decisions are optimized to achieve the best possible outcomes for individual patients while minimizing potential risks. For prescribers, these results highlight the importance of tailoring ARB selection to individual patient needs. For example, olmesartan’s lower RPs for efficacy indicators may make it a suitable choice for patients requiring robust blood pressure control, while its higher RPs for renal and gastrointestinal disorders necessitate careful monitoring in patients with pre-existing conditions in these areas. Such an individualized approach ensures optimal therapeutic outcomes while minimizing potential risks.

## Discussion

The strengths of this study lie in its targeted design and rigorous inclusion criteria, focusing solely on patients prescribed ARBs for hypertension treatment. This methodological approach enhances the validity of the findings by minimizing potential confounding factors, thereby offering a clearer perspective associated with ARBs in hypertension management. Additionally, the comprehensive assessment of seven major ARBs, coupled with the application of Bonferroni correction to address multiple comparisons, ensures statistical robustness and reduces the likelihood of Type I errors.

The results highlight distinct patterns of AEs across different ARBs. For instance, losartan was associated with a higher RP of skin disorders, consistent with previous studies linking it to angioedema [21]. This may be attributed to its effects on the renin-angiotensin system, particularly through angiotensin II type 2 receptor stimulation, which influences vasodilation and vascular permeability [22]. Additionally, losartan's impact on bradykinin metabolism and prostaglandin synthesis may contribute to these effects. Irbesartan exhibited a higher RP for electrolyte disorders, likely due to its pharmacological properties [23]. This underscores the necessity of monitoring electrolyte levels in patients treated with irbesartan. While effective in managing hypertension and preventing cardiovascular and renal complications by blocking AT1 receptors, irbesartan’s mechanism can also disrupt electrolyte homeostasis [24]. Specifically, irbesartan may cause potassium retention due to its inhibition of aldosterone secretion and potentially lead to sodium loss through increased natriuresis. The study corroborates previous studies indicating a low RP for respiratory disorders across all ARBs [25]. Valsartan demonstrated notably higher RP for cardiovascular disorders compared to other ARBs, potentially linked to its widespread use in high-risk cardiovascular patients and its established role in managing conditions such as heart failure and post-myocardial infarction [26]. These findings may reflect valsartan’s pharmacological characteristics, including its selective blockade of AT1 receptors and its effects on reducing left ventricular hypertrophy and improving endothelial function, as well as its frequent application in patients with significant cardiovascular risks [27]. Olmesartan exhibited higher RPs for general, renal, and gastrointestinal disorders compared to other ARBs. Its unique pharmacological properties might explain these findings [28]. Notably, olmesartan-associated enteropathy has garnered attention due to symptoms resembling celiac disease, such as chronic diarrhea and malabsorption [29,30]. This condition appears to be dose-dependent and may have a genetic predisposition, with studies showing a higher prevalence of HLA-DQ2/DQ8 in affected patients. This highlights the need for further investigation into olmesartan’s impact on intestinal permeability and the renin-angiotensin-aldosterone system. Variations in angiotensin receptor binding affinity and half-life among ARBs may contribute significantly to the observed differences in AE profiles. For example, telmisartan’s extended half-life could enhance its efficacy in maintaining blood pressure control but may also increase the risk of dose-dependent AEs such as dizziness or fatigue. Similarly, olmesartan’s higher binding affinity for AT1 receptors might explain its association with gastrointestinal disorders like enteropathy, as its potent receptor blockade could influence intestinal permeability. These findings align with some prior studies but also reveal novel associations that require further investigation to confirm causality and explore underlying mechanisms.

The observed predominance of female patients across all ARBs may reflect sex-related disparities in both pharmacokinetics and pharmacodynamics [15]. Previous studies suggest that females exhibit higher clearance rates for drugs metabolized by CYP3A4, which is involved in the metabolism of several ARBs [14]. Additionally, hormonal influences such as estrogen may modulate drug transporter activity (e.g., P-glycoprotein) and hepatic enzyme expression, potentially affecting ARB metabolism and clearance. These pharmacokinetic differences could contribute to variations in AE profiles between sexes. Furthermore, behavioral factors, including a higher prevalence of hypertension in postmenopausal females and differences in healthcare-seeking behavior, may also play a role in the observed trend. While these findings align with prior pharmacovigilance studies, further investigation is needed to elucidate the interplay between pharmacokinetics, hormonal influences, and AE reporting patterns.

This study has several limitations, primarily stemming from the inherent constraints of the FAERS database. A major limitation is the inability to calculate true incidence rates of AEs within the population using FAERS data, which hinders accurate assessments of the frequency and prevalence of medication-associated AEs. Moreover, the FAERS database is subject to various reporting biases, including underreporting of mild AEs and disproportionate reporting of severe AEs. Additionally, the database contains numerous incomplete or missing entries, further undermining the comprehensiveness and reliability of the data. Patient medical history, apart from hypertension, was largely unavailable, thereby limiting the ability to account for potential confounding factors or pre-existing conditions that may affect the occurrence of AEs. Furthermore, this study excluded patients taking multiple ARBs to isolate the effects of individual ARBs and reduce confounding from polypharmacy. While this approach improves internal validity, it may introduce selection bias by systematically excluding patients with resistant hypertension who often require combination therapy. These patients represent a distinct subgroup with complex clinical profiles that could influence AE reporting patterns. As a result, our findings may not fully generalize to patients on combination therapy. Future studies should include such subgroups to better understand the safety and efficacy of ARB combinations in real-world settings. Additionally, this study did not consider concomitant medications, despite their potential contribution to AE occurrences through polypharmacy. A rigorous analysis of polypharmacy would require pairwise comparisons (e.g., losartan with diuretics vs. losartan without diuretics, valsartan with calcium channel blockers vs. valsartan without calcium channel blockers) while controlling for confounders, a process necessitating extensive subgroup analyses across numerous drug combinations. We believe this level of detail is beyond the focus of this study and could be better addressed in a separate, dedicated investigation.

Despite these limitations, the findings provide valuable insights into the comparative safety profiles of ARBs used in hypertension management. They emphasize the importance of tailoring ARB selection based on individual patient characteristics and potential risk factors. Future studies should validate these results using alternative data sources such as clinical registries, prospective observational studies, or randomized controlled trials to enhance understanding of ARB safety profiles in real-world settings. Such efforts could improve clinical decision-making and reduce AEs associated with hypertension treatment. To improve the usability of FAERS data, future research could explore implementing machine learning approaches for imputing missing data, integrating FAERS with electronic health records to provide more comprehensive patient information, and standardizing reporting formats to improve data quality and consistency. These enhancements could significantly advance our understanding of medication safety and improve patient outcomes in hypertension management.

## Conclusions

This comprehensive analysis of AE profiles among seven ARBs for hypertension management reveals notable variations in safety, particularly in skin disorders, cardiovascular events, and gastrointestinal effects. However, efficacy indicators were assessed indirectly using the FAERS database, and these results should therefore be interpreted with caution. Our findings suggest the potential for tailoring ARB selection based on individual patient risk factors and comorbidities. For example, olmesartan may be avoided in patients with pre-existing gastrointestinal conditions due to its higher RP for gastrointestinal disorders. Head-to-head comparative studies would also be highly beneficial for addressing the limitations of spontaneous reporting data and validating our findings. These studies should ideally focus on both long-term cardiovascular outcomes and AE profiles to provide a comprehensive understanding of ARB effectiveness and safety. Randomized controlled trials comparing individual ARBs could identify specific drugs that are more suitable for high-risk patients, such as avoiding olmesartan in those with gastrointestinal issues. Such studies would help refine personalized treatment strategies by linking drug selection to patient-specific risks and comorbidities. Leveraging electronic health records (EHRs) and clinical registries offers significant potential for advancing research on ARB safety and efficacy. However, several challenges must be acknowledged. Heterogeneity in data collection methods across institutions can lead to inconsistencies in patient demographics, clinical outcomes, and AE reporting. Coding practices may vary, introducing discrepancies in how AEs are classified and recorded. Additionally, confounding factors such as polypharmacy or underlying comorbidities may complicate the interpretation of observational data. Addressing these challenges requires standardized protocols for data collection and integration, as well as advanced statistical methods to account for confounders. Despite these obstacles, the integration of EHRs with pharmacovigilance databases could significantly enhance the quality and applicability of real-world evidence. Future research should focus on exploring specific patient subgroups, such as those with renal disease or diabetes, to better understand how comorbidities influence ARB safety and efficacy. By narrowing the scope to specific ARBs or AEs, it may be possible to reduce sample size requirements to a feasible level, enabling prospective clinical trials to directly compare the relative safety and efficacy of individual ARBs. These efforts could ultimately lead to more personalized treatment strategies for hypertension management and improved patient outcomes.

## Appendices

Appendix A

We processed patient background variables prior to statistical analysis as follows: The {sex} variable, labeled {gndr_cod} between 2004Q1 and 2014Q2 and {sex} between 2014Q3 and 2024Q4, was treated uniformly. Entries other than "M" or "F," or those missing, were classified as unknown. The {age} variable, reported in various units (years, decades, months, weeks, or days), was standardized to years. We employed specific conversion methods: decade units were multiplied by 10 and increased by 5; month units were divided by 12; week units by 365.25 / 7; and day units by 365.25, all rounded down to whole numbers. The {weight} variable, predominantly recorded in kilograms, was converted from pounds when necessary by multiplying by 0.454. The {reporter_country} variable was replaced with "COUNTRY NOT SPECIFIED" for unknown entries.

Appendix B

**Table 4:** Generic and brand names of seven angiotensin receptor blockers

Generic name	Brand name
Losartan	Cozaar
Valsartan	Diovan
Irbesartan	Aprovel, Avapro
Candesartan	Atacand, Blopress
Telmisartan	Micardis
Olmesartan	Benicar
Azilsartan	Edarbi

Appendix C

**Table 5:** Preferred terms used to define patients with hypertension

Preferred terms
Accelerated hypertension, diastolic hypertension, essential hypertension, hypertension, hypertensive cardiomegaly, hypertensive cardiomyopathy, hypertensive crisis, hypertensive emergency, hypertensive end-organ damage, hypertensive heart disease, hypertensive nephropathy, hypertensive urgency, labile hypertension, malignant hypertension, malignant hypertensive heart disease, neurogenic hypertension, orthostatic hypertension, prehypertension, secondary hypertension, supine hypertension, systolic hypertension, withdrawal hypertension

Appendix D

**Table 6:** Categorization of preferred terms Preferred terms are presented exactly as they appear in the  FAERS to maintain consistency with the original database and ensure searchability. FAERS: United States Food and Drug Administration Adverse Event Reporting System

Adverse event category	Preferred terms
Skin disorders	Angioedema, erythema, hyperhidrosis, peripheral swelling, pruritus, and rash
Electrolyte disorders	Hyperkalemia, hypokalemia, and hyponatremia
Respiratory disorders	Cough, dyspnea, and interstitial lung disease
Neurological disorders	Anxiety, depression, dizziness, headache, insomnia, memory impairment, paresthesia, somnolence, and tremor
Cardiovascular disorders	Arrhythmia, blood pressure decreased, blood pressure increased, cardiac failure, chest pain, hypotension, myocardial infarction, orthostatic hypotension, palpitations, and syncope
General disorders	Asthenia, fatigue, malaise, pyrexia, and weight decreased
Renal disorders	Acute kidney injury, blood creatinine increased, chronic kidney disease, renal failure, and renal impairment
Gastrointestinal disorders	Abdominal pain, constipation, diarrhea, dyspepsia, gastritis, gastroesophageal reflux disease, nausea, sprue-like enteropathy, and vomiting
Efficacy indicators	Blood pressure inadequately controlled, drug ineffective, and hypertension

Appendix E

**Table 7:** Cross-tabulation of adverse event categories with angiotensin receptor blockers showing significant ROR and aROR values, compared to the reference angiotensin receptor blocker <1: Indicates that both ROR and aROR are significantly <1 compared to the reference angiotensin receptor blocker; >1: Indicates that both ROR and aROR are significantly >1 compared to the reference angiotensin receptor blocker; N.S.: Indicates that at least one of ROR or aROR is not significant compared to the reference angiotensin receptor blocker. aROR: adjusted reporting odds ratio; ROR: reporting odds ratio

	Losartan	Valsartan	Irbesartan	Candesartan	Telmisartan	Olmesartan	Azilsartan
Skin disorders	>1	Reference	N.S.	N.S.	N.S.	<1	N.S.
Electrolyte disorders	>1	Reference	>1	>1	>1	>1	N.S.
Respiratory disorders	N.S.	Reference	N.S.	N.S.	N.S.	<1	N.S.
Neurological disorders	N.S.	Reference	<1	N.S.	N.S.	N.S.	N.S.
Cardiovascular disorders	<1	Reference	<1	<1	<1	<1	N.S.
General disorders	<1	Reference	<1	N.S.	<1	>1	N.S.
Renal disorders	N.S.	Reference	>1	N.S.	N.S.	>1	N.S.
Gastrointestinal disorders	N.S.	Reference	N.S.	N.S.	N.S.	>1	N.S.
Efficacy indicators	N.S.	Reference	<1	N.S.	N.S.	N.S.	N.S.
Skin disorders	>1	N.S.	Reference	N.S.	N.S.	<1	N.S.
Electrolyte disorders	N.S.	<1	Reference	<1	N.S.	N.S.	N.S.
Respiratory disorders	>1	N.S.	Reference	>1	N.S.	N.S.	N.S.
Neurological disorders	>1	>1	Reference	>1	N.S.	N.S.	N.S.
Cardiovascular disorders	N.S.	>1	Reference	>1	N.S.	>1	N.S.
General disorders	N.S.	>1	Reference	N.S.	N.S.	>1	N.S.
Renal disorders	<1	<1	Reference	N.S.	<1	N.S.	N.S.
Gastrointestinal disorders	N.S.	N.S.	Reference	N.S.	N.S.	>1	N.S.
Efficacy indicators	N.S.	>1	Reference	>1	N.S.	N.S.	N.S.
Skin disorders	>1	N.S.	N.S.	Reference	N.S.	N.S.	N.S.
Electrolyte disorders	N.S.	<1	>1	Reference	N.S.	N.S.	N.S.
Respiratory disorders	N.S.	N.S.	<1	Reference	N.S.	<1	N.S.
Neurological disorders	N.S.	N.S.	<1	Reference	N.S.	N.S.	N.S.
Cardiovascular disorders	N.S.	>1	<1	Reference	N.S.	N.S.	N.S.
General disorders	N.S.	N.S.	N.S.	Reference	<1	>1	N.S.
Renal disorders	N.S.	N.S.	N.S.	Reference	N.S.	N.S.	N.S.
Gastrointestinal disorders	N.S.	N.S.	N.S.	Reference	N.S.	>1	N.S.
Efficacy indicators	N.S.	N.S.	<1	Reference	N.S.	N.S.	N.S.
Skin disorders	>1	N.S.	N.S.	N.S.	Reference	<1	N.S.
Electrolyte disorders	N.S.	<1	N.S.	N.S.	Reference	N.S.	N.S.
Respiratory disorders	N.S.	N.S.	N.S.	N.S.	Reference	<1	N.S.
Neurological disorders	N.S.	N.S.	N.S.	N.S.	Reference	N.S.	N.S.
Cardiovascular disorders	N.S.	>1	N.S.	N.S.	Reference	N.S.	N.S.
General disorders	N.S.	>1	N.S.	> 1	Reference	>1	N.S.
Renal disorders	N.S.	N.S.	>1	N.S.	Reference	>1	N.S.
Gastrointestinal disorders	N.S.	N.S.	N.S.	N.S.	Reference	>1	N.S.
Efficacy indicators	N.S.	N.S.	N.S.	N.S.	Reference	N.S.	N.S.
Skin disorders	>1	>1	>1	N.S.	>1	Reference	N.S.
Electrolyte disorders	N.S.	<1	N.S.	N.S.	N.S.	Reference	N.S.
Respiratory disorders	>1	>1	N.S.	>1	>1	Reference	N.S.
Neurological disorders	N.S.	N.S.	N.S.	N.S.	N.S.	Reference	N.S.
Cardiovascular disorders	<1	>1	<1	N.S.	N.S.	Reference	N.S.
General disorders	<1	<1	<1	<1	<1	Reference	N.S.
Renal disorders	<1	<1	N.S.	N.S.	<1	Reference	N.S.
Gastrointestinal disorders	<1	<1	<1	<1	<1	Reference	<1
Efficacy indicators	N.S.	N.S.	N.S.	N.S.	N.S.	Reference	N.S.
Skin disorders	N.S.	N.S.	N.S.	N.S.	N.S.	N.S.	Reference
Electrolyte disorders	N.S.	N.S.	N.S.	N.S.	N.S.	N.S.	Reference
Respiratory disorders	N.S.	N.S.	N.S.	N.S.	N.S.	N.S.	Reference
Neurological disorders	N.S.	N.S.	N.S.	N.S.	N.S.	N.S.	Reference
Cardiovascular disorders	N.S.	N.S.	N.S.	N.S.	N.S.	N.S.	Reference
General disorders	N.S.	N.S.	N.S.	N.S.	N.S.	N.S.	Reference
Renal disorders	N.S.	N.S.	N.S.	N.S.	N.S.	N.S.	Reference
Gastrointestinal disorders	N.S.	N.S.	N.S.	N.S.	N.S.	> 1	Reference
Efficacy indicators	N.S.	N.S.	N.S.	N.S.	N.S.	N.S.	Reference
